# Emerging Diagnostic Tools to Decide When to Discontinue Nucleos(t)ide Analogues in Chronic Hepatitis B

**DOI:** 10.3390/cells9020493

**Published:** 2020-02-20

**Authors:** Margarita Papatheodoridi, George Papatheodoridis

**Affiliations:** 1 Institute of Liver and Digestive Health, Royal Free Hospital, University College of London, London NW3 2QG, UK; margarita.gpap@gmail.com; 2 Department of Gastroenterology, Medical School of National and Kapodistrian University of Athens, General Hospital of Athens “Laiko”, 11527 Athens, Greece

**Keywords:** discontinuation, nucleos(t)ide analogues, hepatitis b, HBsAg, HBcrAg, relapse, retreatment, remission, HBsAg loss

## Abstract

The aim of this review is to outline emerging biomarkers that can serve as diagnostic tools to identify non-cirrhotic chronic hepatitis B (CHB) patients who could safely discontinue nucleos(t)ide analogues (NAs) before HBsAg loss. Regarding possible predictors of post-NAs outcomes, a number of studies have evaluated numerous factors, which can be categorised in markers of hepatitis B virus (HBV) activity, markers of host immune response and markers of other patient characteristics. In clinical practice, the most important question for patients who discontinue NAs is to differentiate those who will benefit by achieving HBsAg loss or at least by remaining in remission and those who will relapse requiring retreatment. Most of the discontinuation studies so far came from Asian and only few from European populations and examined the rates and predictors of post-NA virological and/or combined relapses or HBsAg loss. To date, there is still controversy about predictors of post-NA relapses, while only HBsAg serum levels at NA discontinuation seem to be the most robust predictive marker of the probability of subsequent off-treatment HBsAg seroclearance. Newer viral markers such as HBV RNA and hepatitis B core-related antigen seem promising, but further research is required.

## 1. Introduction

Chronic hepatitis B (CHB) remains a major health problem worldwide [1] and, despite progress that has been made in the last decades, long-term or lifelong treatment with nucleos(t)ide analogues (NAs) is usually required [2,3,4]. This is due to the fact that, although NAs manage to effectively suppress viral replication by blocking reverse transcriptase of hepatitis B virus (HBV), functional cure defined by hepatitis B surface antigen (HBsAg) loss is not frequently achieved, while eradication of HBV from the host liver cells is not feasible with the current treatment options [2,3,4].

However, the latest recommendations from European, US and Asian liver associations (EASL, AASLD, APASL) suggest that NAs cessation may be considered in selected CHB patients before HBsAg loss [3,4,5,6]. Regarding hepatitis B e antigen (HBeAg)-positive CHB patients, there is significant concordance among guidelines from the different associations: non-cirrhotic patients with HBeAg-positive CHB can discontinue NAs when they have completed at least 12–36 months of consolidation therapy after seroconversion of HBeAg to anti-HBe and HBV DNA undetectability [3,4,5,6]. For HBeAg-negative CHB patients, the recommendations are controversial perhaps due to the heterogeneity of post-NAs outcomes [7,8,9,10]. The Asian guidelines recommend that NAs cessation can be attempted after at least 2 years of effective therapy in non-cirrhotic patients with HBeAg-negative CHB if serum HBV DNA remains undetectable on three separate occasions 6 months apart [5,6]. The latest EASL recommendations included the possibility of stopping NAs and suggested that NA treatment may be discontinued in selected non-cirrhotic HBeAg-negative CHB patients who have remained under on-therapy virological suppression for at least 3 years and will comply to close post-NAs follow up [3], but AASLD has not included a similar recommendation for HBeAg-negative CHB patients [4].

CHB patients with compensated cirrhosis are considered a patient subgroup at high risk for liver decompensation and hepatic failure in case of HBV flares following NAs cessation. Thus, NA discontinuation may be considered in patients with cirrhosis who will remain under strict surveillance only in the Asian recommendations and mainly for countries with very limited resources [5], but not in the other two major scientific associations [3,4].

Even for the non-cirrhotic CHB patients in whom NA discontinuation is considered safe under the aforementioned conditions [7], clinical experience is still restricted in a few parts of the world and this renders the decisions quite challenging for the physicians. Added to that, the current recommendations are not as practical and specific as needed and their usefulness is limited by the diversity of patient outcomes after NAs discontinuation. To this end, various studies have tried to evaluate possible predictive markers of patients’ post-NA outcomes, which could be broadly classified into virological relapse usually defined as HBV DNA > 2000 IU/mL, clinical relapse defined as HBV DNA > 2000 IU/mL and elevated alanine aminotransferase (ALT) levels [usually more than twice the upper limit of normal (ULN)] and HBsAg loss.

This review aims to summarize such emerging biomarkers that could serve as useful diagnostic tools to safely guide non-cirrhotic CHB patients’ selection for NAs discontinuation before HBsAg loss.

## 2. Diagnostic Tools for Identifying On-Therapy CHB Patients Who May Benefit from NAs Discontinuation before HBsAg LOSS

### 2.1. Standard HBV Virological Markers

#### 2.1.1. HBeAg

The most useful and well known biomarkers that guide clinical decisions regarding the management of CHB patients are the markers of HBV activity. To begin with, HBeAg is used to define the stages of chronic HBV infection but it also contributes in defining an endpoint of NAs discontinuation [3,5]. Therefore, different duration of treatment and on-treatment virological suppression may be required in HBeAg-positive and HBeAg-negative CHB patients before NA cessation can be considered. In particular, at least 12 months of consolidation NA therapy after HBeAg seroconversion and HBV DNA undetectability for HBeAg-positive and at least 36 months of effective NA therapy for HBeAg-negative CHB patients are recommended before NA discontinuation according to the European recommendations [3].

Still after NA cessation, these patient subgroups seem to have distinct outcomes. The results from a systematic review showed that the pooled durable rates of virological remission at 12 and 24 months after NA discontinuation were 63% and 53% in pretreatment HBeAg-positive and 44% and 31% in HBeAg-negative CHB patients (*p* = 0.064) [7]. On the other hand, a recent randomized controlled trial reported that 61% of pretreatment HBeAg-positive versus 22% of HBeAg-negative CHB patients required retreatment after NAs discontinuation (*p* = 0.01) [11]. Given that, it is apparent that NAs discontinuation studies should examine the outcomes and predictors for these subgroups of CHB patients separately. However, this is not the case for a number of studies mainly from Asia that have attempted to provide predictors in cohorts with both initially HBeAg-positive and HBeAg-negative patients [12,13]. Therefore, when looking for useful and reliable markers for NAs discontinuation, the cohort used for the development of these markers should be considered in order to estimate the applicability of the results in the population of interest. The main characteristics of studies including initially HBeAg-positive or HBeAg-negative CHB patients who discontinued NAs before HBsAg loss and reported predictors of post-NA virological relapse, clinical relapse and HBsAg loss are shown in Table 1, Table 2 and Table 3, respectively.

#### 2.1.2. HBsAg

The second most important and valuable viral marker is HBsAg. To date, HBsAg quantification (qHBsAg) in serum is performed on widely available instruments used for routine serology with an automated method which is considered highly reproducible [40,41].

HBeAg and HBsAg represent the main tools which define the subgroups of patients who could stop NAs. First, it is quite clearly stated in all currently available international guidelines [3,4,5] that patients who achieve HBsAg seroconversion can safely stop NAs, since this is considered the stage of functional cure. Additionally, it is also evident that patients who maintain HBeAg seropositivity should not stop treatment. Therefore, when we evaluate markers which would help us define the optimal time point for treatment cessation, we refer to patients who were HBeAg positive or negative before therapy and remain HBeAg negative and HBsAg positive under therapy; this is applied to all of the reviewed studies.

Regarding HBsAg-positive patients’ outcomes after NAs discontinuation, serum HBsAg levels are a tool of great importance [42]. A number of studies have evaluated HBsAg levels during NAs treatment [14,16,18,22,37,38,43,44] or most commonly at the end of treatment (EOT) as possible predictors [14,15,16,17,18,21,22,23,24,26,27,28,29,30,32,34,35,36,37,38,39,44]. This idea was first suggested in the pivotal study of Hadziyannis et al., where HBsAg levels at EOT were associated with subsequent HBsAg loss in 33 non-cirrhotic HBeAg-negative CHB patients [36]. Since then, this finding has been confirmed in a number of studies, where it has been reported that lower EOT qHBsAg levels are associated with higher probability of off-NAs HBsAg loss [14,15,21,22,37,38,39], while higher EOT qHBsAg levels may be associated with higher probability of virological [14,16,18,19,21,22,24,27,28,29] or clinical relapses in other cohorts [16,27,28,32,34,35,44]. Of note, there has been no negative study assessing the association between EOT HBsAg and off-NAs HBsAg loss. In contrast, the associations of EOT qHBsAg with virological and/or clinical relapses have not been confirmed in all studies including either HBeAg-positive [14,16], HBeAg-negative [23,30], or both HBeAg-positive and HBeAg-negative CHB patients [17,26].

Pretreatment qHBsAg levels have not been associated with patient outcomes in any [14,18,22,30,33,44] but one study assessing this marker [16]. In that study, Chen et al. showed that high pretreatment qHBsAg levels (>1000 IU/mL) were independently associated with increased risk of virological relapse defined as HBV DNA >2000 IU/mL in initially HBeAg-positive patients who discontinued NAs [16]. Furthermore, declining qHBsAg levels during or after treatment have also been found to predict subsequent HBsAg loss [16,38,39] or off-therapy virological remission [37]. Therefore, studying qHBsAg kinetics has gathered researchers’ interest, since it seems that serial measurements of qHBsAg can be useful in guiding clinical decisions and managing these patients. The abovementioned remarks are depicted in detail in Table 1, Table 2 and Table 3.

#### 2.1.3. HBV DNA

Serum viral load, which is measured via serum HBV DNA quantification, has been assessed in various settings as a tool for treatment decisions in CHB patients, but in respect to patient management after NAs discontinuation, only few studies showed that there is association between higher pretreatment HBV DNA levels and post-NAs relapses [17,18,21,22,29,30,32] or HBsAg loss [14,33]. On the other hand, negative results were provided by a number of other cohorts that have examined the association between pretreatment serum HBV DNA levels and virological relapses [14,16,17,23,24,25,44], clinical relapses [23,31,33,34,35,44,45] or HBsAg loss [14,22,36,37,39].

One study showed that delayed suppression of HBV DNA during treatment was an independent predictor of post-NAs relapse [24], but this has not been evaluated in any other cohort. In a systematic review [7], the probability of durable off-NA(s) virological remission was found to be significantly higher in HBeAg-negative CHB patients who remained in virological remission under NA(s) for >24 than ≤24 months. Thus, duration of on-therapy virological remission was suggested to represent a critical factor associated with the probability of durable off-NA(s) virological remission in this setting [7,20]. Finally, it has been once supported that detectable HBV DNA levels at one month after NA discontinuation are associated with subsequent clinical relapses [31], a finding that has not been confirmed in our study which was the only one that has also assessed this association [23]. Therefore, given the heterogeneity of results in the different studies, HBV DNA does not seem to be a reliable marker for selecting CHB patients eligible for NAs discontinuation before HBsAg loss.

Similarly, low clinical value seems to be offered by the identification of HBV genotype in patients who stop NAs, since there is no proven association between any specific genotype and post-NA outcomes in almost any study so far. Only, in the initially HBeAg-positive patients of the cohort of Chen et al. genotype C was associated with increased risk of virological and/or clinical relapses [16]. However, this finding is not enough to support any potential utility of examining HBV genotype in patients who discontinue NAs. On the contrary, it can be assumed that predictors and outcomes from various cohorts after NAs discontinuation may be of value in different patient populations irrespective of their HBV genotype.

Interestingly, very recent data from two cohorts highlighted the importance of serum titers of antibody against hepatitis B core antigen (anti-HBc) as a predictive marker for clinical relapses. Tseng et al. [28] showed that there is a trend for inverse association between anti-HBc titers and the risk of clinical relapses. More recently, Chi et al. reported 4-year clinical relapse rate of 21% in patients with EOT anti-HBc levels ≥ 1000 IU/mL in comparison to 85% of patients with levels < 100 IU/mL (*p* < 0.001) [35]. Apparently, future research is needed to define whether there is some clinical utility in this finding.

### 2.2. Newer HBV Virological Markers

Emerging viral markers, such as hepatitis B core-related Antigen (HBcrAg) and serum HBV RNA levels have drawn researchers’ attention lately. Particularly, HBcrAg was reported to be strongly correlated with intrahepatic HBV cccDNA and was considered as a tool that could be used to monitor disease progression [46] as well as to identify patients in remission more accurately than qHBsAg [47]. More than a decade ago, results from a Japanese cohort supported that HBcrAg can identify patients who remain in remission after lamivudine discontinuation [48]. Later, Jung et al. showed that EOT HBcrAg > 3.7 log IU/mL was independently associated with more than three-times higher risk of virological relapse in HBeAg-negative CHB patients. Hsu et al. also showed that HBcrAg was independently associated with relapses after NAs discontinuation and they additionally combined it with other predictive markers to create a predictive score model, SCALE-B score [34]. Finally, results from the DARING-B study confirmed the predictive value of HBcrAg for prediction of serious relapses that require retreatment in Caucasian HBeAg-negative CHB patients who discontinued entecavir (ETV) or tenofovir disoproxil fumarate (TDF) therapy, showing that detectable EOT HBcrAg levels could rule out the probability of HBsAg loss and were independently associated with more than 3-fold increased risk of retreatment [39].

Serum HBV RNA levels, which seem to correlate with intrahepatic HBV cccDNA transcriptional activity [40], have been less studied in the setting of NAs discontinuation. It is suggested, though, that using HBV RNA levels in combination with HBV DNA levels can predict the risk of off-therapy relapses [49], whereas more recent data supported that undetectability of both serum HBV DNA and HBV RNA at EOT was associated with post NAs remission [45]. Yet, further research is needed to answer whether these promising markers may be used in clinical practice in this setting.

### 2.3. Markers of Host Immune Activity

Moving one step further from the viral markers, studying the mechanisms of host immune activity in patients who discontinue NAs seems to be of great value, although immune biomarkers might be used for research purposes only for the time being.

To this end, a small yet very interesting study of Kranidioti et al. found a distinct immune signature in HBeAg-negative CHB patients who remained in off-treatment remission for 3 years after NAs cessation and suggested that RNA levels of interferon gamma (IFNg), interleukin 8 (IL-8), Fas Ligand (FASLG) and carbon tetrachloride (CCL4) from peripheral blood mononuclear cells (PBMCs) might be used as potential biomarkers in this setting [50].

The study of Rivino et al. also supported that a distinct subpopulation of HBV-specific cells might serve as a biomarker for NAs discontinuation, since they showed that post-NAs remission correlated with the presence of HBV core and polymerase-specific T cells that were contained within the ex vivo programmed death 1 (PD-1) population during NAs viral suppression. According to the authors, this also underlines the value of T-cell exhaustion markers that should be further evaluated in this setting [51].

On the other side, distinct T-cell phenotype was detected in CHB patients under long-term NA therapy and was only slightly altered after treatment discontinuation, according to Rinker et al. [52]. In that study in vitro peptide stimulated HBV-specific T-cell responses were induced in several patients after NAs cessation, while blocking of programmed death-ligand 1 (PD-L1) further enhanced HBV-specific T cell responses. Therefore, virological relapses may trigger the immunological environment that enhances the responsiveness of HBV-specific T-cells in vitro [52].

The same team suggested that other cell types might also be involved in the immune response after NAs discontinuation in CHB patients. Special natural killer (NK) cell compartment has been revealed by unsupervised stochastic neighbour embedding analysis; increased activity of NK cells correlated with post-NAs biochemical flares and was particularly enhanced in patients experiencing subsequent HBsAg loss [53].

Furthermore, various plasma cytokines and growth factors have been assessed as biomarkers indicating the activation of immune responses. A small prospective study that included 15 HBeAg-negative CHB patients showed that off treatment virological flares were associated with induction of plasma tumour necrosis factor α (TNF-α), interleukin 10 (IL-10), interleukin 12p70 (IL12p70) and interferon inducible protein 10 (IP10). These markers significantly increased in the first month after NAs discontinuation, while IP10 peaked at month 2 [54]. We also presented similar kinetics of IP10 levels in a larger cohort of 57 HBeAg-negative CHB patients showing that IP10 levels at month 1 after NA discontinuation were independently associated with subsequent HBsAg loss probably suggesting that early untreated post NAs immune activity might contribute to subsequent functional cure [39]. On the other hand, Wong et al. showed that IP10 at the time of HBsAg seroclearance was associated with HBsAg loss, while various other serum cytokines that were examined (interleukin (IL)-2, IL-3, IL-4, IL-7, IL-9, IL-10, IL-12, IL-15, IL-21 interferon-c, TNFa, granulocyte macrophage colony-stimulating factor (GM-CSF)) were not [55]. However, IP10 levels at 3 years before HBsAg loss were not associated with the outcome and thus the predictive utility of this finding seems limited.

Driven by the concept that off-treatment immunological response may lead to immune control and functional cure, some studies have supported that patients who suffered virological rebounds and/or biochemical flares and were not retreated had higher probability of subsequent HBsAg loss [56]. In this context, Liaw et al. has supported the “stop’n’watch” strategy, based on which off-NAs flares can be left untreated yet closely followed, since there is high probability that they may be transient and lead to remission and/or HBsAg loss [57]. However, although universal reinitiation of NAs should be avoided in patients with transient virological relapses, it should always be underlined that there is no strong evidence to date to support that severe flares are beneficial and more importantly safe enough to be left untreated. Thus, careful monitoring is warranted to reinitiate timely treatment in order to prevent harmful consequences in patients with uncontrolled relapses [58,59].

### 2.4. Patient Characteristics

There are suggestions that some basic patient characteristics may serve as markers for the physician to select the CHB patients who would be eligible for safe treatment discontinuation, even before they start the long-term NA therapy. However, there is no concordance among the various studies’ results (Table 1, Table 2 and Table 3) and therefore relevant conclusions need to be drawn with caution.

There are five studies suggesting that older age is associated with post-NAs virological relapses [14,16,17,18,29] and one with clinical relapses [34]. In some of these studies, patients older than 40 or 50 years old were reported to be at higher risk of virological relapses [16,17,18]. On the contrary, more than 10 of the studies do not support that age is a predictive factor of off-treatment relapses [15,22,23,24,25,26,28,30,31,32,33,35,44]. In addition, age was not associated with HBsAg loss in any study assessing such an association [14,22,33,36,39].

In one study [14], male sex was independently associated with higher risk of virological relapses, but such an association has not been confirmed in any other cohort (Table 1) nor has it been shown any association of patients’ sex with clinical relapse (Table 2) or HBsAg loss (Table 3).

Among the liver function tests, levels of alanine aminotransferase (ALT) before treatment were the most commonly examined marker as possible predictor of post-NA relapses or HBsAg loss (Table 1, Table 2 and Table 3). Nevertheless, most of the studies did not show any association between pretreatment ALT and the probability of post-NA virological [14,15,16,18,22,23,24,25,44], clinical relapse [16,23,34,35] or HBsAg loss [14,15,22,33,39]. There were, though, two studies suggesting that higher pretreatment ALT levels were associated with higher probability of HBsAg loss [14,36] and four studies showing that they were associated with lower risk of virological [15] and clinical relapse [28,31,33]. This association, although not widely confirmed, seems rational and might be explained by the fact that if a patient had high ALT levels before treatment, which would suggest strong immune response and absence of the “T-cell exhaustion” phenomenon, they may still host functional HBV-specific T-cells. That would potentially enable the activation of an immune response which could lead to post-NAs remission or functional cure.

In addition, there are interesting recent findings from few studies suggesting that the choice of NA might affect the risk of relapse after treatment discontinuation. A study from Germany [26] and two studies from Asia [27,34] reported that TDF use can increase the risk of or shorten the timing of post-NA relapse [27]. However, TDF use was not found to independently affect the timing of post-NA relapses in another study including Greek and Taiwanese CHB patients [60]. Although further research is needed to validate these results, whether TDF users carry greater and/or more rapid risk of post-NAs relapse is undoubtedly a question of interest.

Finally, even if cirrhotic patients are excluded from the “stopping-NAs” strategy, since this patient subgroup is more likely to face an imminent peril with uncontrolled HBV flares after treatment discontinuation [3,7], the severity of pretreatment fibrosis may be still a predictor of post-NA relapse. According to our recent prospective study, more severe pretreatment fibrosis (F3 Metavir score) was associated with worse outcome and more specifically with increased risk of retreatment in non-cirrhotic HBeAg-negative CHB patients who discontinued NA therapy [23]. Facing this from a different point of view, the majority of that study’s patients with mild to moderate fibrosis remained without retreatment at least 18 months after NA discontinuation [23].

## 3. Discussion and Conclusions

Long-term, perhaps indefinite, NA therapy is usually recommended for CHB patients, particularly for HBeAg-negative cases treated in Western countries. Thus, discontinuation of NAs in patients with HBeAg-positive and especially HBeAg-negative CHB who remain HBsAg positive is still considered a controversial approach by many, at least Western, experts, although it has been included in the Asian guidelines for >7 years [6] and in the European guidelines for >2 years [3].

In clinical practice, the most important decision for patients who discontinue NAs is to distinguish between those who will have a benefit by achieving HBsAg loss or at least remaining in remission and those who will relapse and require retreatment. Although the first relevant studies were published almost a decade ago, post-NAs remission has been used as an endpoint in only two old studies [37,43], while the majority of recent studies have not examined predictors of sustained remission and preferred setting stronger endpoints such as virological relapses and/or HBsAg loss.

Regarding HBsAg loss, there has been only one reliable predictor, EOT qHBsAg levels, which were significantly associated with the probability of subsequent HBsAg clearance in all nine relevant studies. The most commonly proposed HBsAg levels cut-off for optimal predictability was 100 IU/mL, as shown in Table 3. Thus, based on the existing data, patients who fulfil the Asian and/or the European criteria for NAs discontinuation and have HBsAg levels below 100 IU/mL can safely discontinue treatment having a high probability of achieving HBsAg loss, which currently defines “functional cure” [39,42].

In the 24 studies evaluating predictors of post-NAs virological relapses (Table 1), the most common predictor was higher EOT HBsAg levels, as validated in 13 of 18 studies, and the second most common was older age, which however was confirmed in only nine of 19. Regarding clinical relapses, there is less data and even less concordance (Table 2). In 12 studies assessing this endpoint, EOT HBsAg levels were the most common predictor as presented in seven of 10 studies, while age was again the second most common predictor according to the results of only four of 11 studies.

Since the majority of CHB patients who discontinue NAs develop virological relapses and a proportion of them experience biochemical relapses and occasionally ALT flares, it is reassuring that prompt retreatment can reintroduce biochemical and virological remission [7]. At the same time, HBsAg loss develops in progressively increasing proportions of CHB patients who discontinue NAs and rash retreatment may prevent the development of such a favourable outcome [7,59].

In conclusion, the identification of factors that can reliably predict the outcomes of CHB patients who discontinue NAs is quite important. Unfortunately, although there have been many studies trying to identify predictors of post-NA relapses, accurate predictors have not been reliably identified so far. The probability of HBsAg loss after NA discontinuation is high in patients with qHBsAg levels ≤ 100 IU/mL and much lower in patients who have qHBsAg levels > 100 and particularly > 1000 IU/mL, but large proportions of these patients are not going to have biochemical relapses and will not require retreatment. Newer markers like serum HBcrAg and HBV RNA levels are currently under investigation as predictors of post-NAs outcomes and seem to be the most promising ones for optimizing prediction in this setting. However, the outcome of patients who discontinue NAs cannot be reliably predicted today.

## Figures and Tables

**Table 1:** Predictors of virological relapse after discontinuation of nucleos(t)ides analogues (NAs) in studies including chronic hepatitis B (CHB) patients who remained HBsAg positive at the end of therapy (EOT). Predictors are provided separately for pretreatment HBeAg-positive patients who achieved HBeAg seroconversion and pretreatment HBeAg-negative patients, when available, or mixed patients when separate analyses were not provided.

Study	Patients, n/Cirrhosis	qHBsAg at EOT, IU/mL	Pretreatment HBV DNA, IU/mL	Age, Years	Pretreatment ALT	Sex	Treatment Duration	TDF vs. ETV	Other Markers Tested
**Pretreatment HBeAg-positive patients**									
Chen, 2014 [14]	83/12	No	No	Yes	No	No	No	-	Cirrhosis, Bilirubin, HBV genotype, Pretreatment/on-treatment HBsAg, Remission Duration: No
Chi, 2015 [15]	35/14	-	-	No	Yes	-	Yes	-	Cirrhosis, Asian ethnicity: No
Chen, 2015 [16]	83/6	No	No	Yes, c.o.: 40	No	No	No	-	Genotype C, Pretreatment qHBsAg: Yes; Cirrhosis, Bilirubin, Prior LAM, On-treatment/decline of HBsAg levels: No
Jung, 2016 [17]	45/15	-	Yes; c.o.: 2 × 10^6^	Yes, c.o.: 40	-	-	-		ETV: no
Qiu, 2016 [18]	112/0	Yes	Yes	Yes, c.o.: 50	No	No	-	-	qHBsAg at HBeAg seroconversion: Yes; Genotype, Pretreatment HBsAg, treatment duration until HBV DNA undetectability: No
Chen, 2018 [19]	39/0	Yes; c.o.: 200	No	No	No	No	No	-	Bilirubin, Genotype, Pretreatment qHBsAg, qHBsAg decline during treatment: No
**Pretreatment HBeAg-negative patients**									
Chen, 2014 [14]	105/32	Yes; c.o.: 200	No	Yes	No	No	No	-	Cirrhosis, Bilirubin, HBV genotype, pretreatment/on-treatment HBsAg, Remission Duration: No
Chi, 2015 [15]	59/13	-	-	No	No	-	Yes	-	Cirrhosis: Yes; Asian ethnicity: no
Chen, 2015 [16]	169/29	Yes; c.o.: 150	No	Yes; c.o.: 55	No	No	No	-	Cirrhosis, ALT, Genotype, Pretreatment/on-treatment/decline of HBsAg levels: No
Jun, 2016 [20]	58	-	-	-	-	-	Yes		Early virological response: Yes
Jung, 2016 [17]	68/18	No	No	Yes; c.o.: 40	-	-	-	-	HBcrAg at EOT:Yes, c.o.: 3.7 log IU/mL
Hung, 2017 [21]	73/73	Yes; c.o.: 300	Yes	-	-	-	-	-	
Yao, 2017 [22]	119*/-	Yes	Yes	No	No	No	Yes	(ETV>LAM)	Cirrhosis, Pretreatment qHBsAg: Yes; Bilirubin, Genotype, qHBsAg decline during treatment: No
Chen, 2018 [19]	90/0	Yes; c.o.: 80	No	No	No	No	No	-	Bilirubin, Genotype, qHBsAg at baseline or decline during treatment: No
Papatheodoridis, 2018 [23]	57/0	No	No	No	No	No	No	-	Body mass index, Alcohol consumption, Pretreatment AST, albumin, white blood cells, platelets, fibrosis: No; Liver stiffness at EOT et al.: No
**Pretreatment HBeAg-positive or HBeAg-negative patients**									
Liang, 2011 [24]	84/0	Yes	No	No	No	No	-	-	HBeAg status: No; LAM resistance; delayed suppression of HBV DNA during therapy: Yes
Ridruejo, 2013 [25]	33/-	-	No	No	No	No	No	-	Cirrhosis: No;
Chen, 2014 [14]	188/44	Yes	No	Yes	No	Yes	No	-	Cirrhosis, Bilirubin, HBeAg; HBV genotype, HBsAg pretreatment/on-treatment, Remission duration: No
Sohn, 2014 [12]	95/44	-	-	-	-	-	Yes	-	HBeAg, NA selection: No
Park, 2016 [13]	103/-	-	-	-	-	-	-	-	Time to on-ETV virological remission, Bilirubin at EOT: Yes
Honer zu Siederdissen, 2018 [26]	220/0	No	-	No	-	No	-	Yes	ALT EOT, HBeAg: No
Su, 2018 [27]	100/-‘	Yes, c.o.: 50, 200 IU/mL	-	-	-	-	-	Yes	Pretreatment HBeAg, CTLA4-nonGG: Yes; EOT Anti-HBc, rs3077 non-GG, rs9277535 non-GG, NTCP non-GG: No
Tseng, 2018 [28]	82/0	Yes	-	No	-	-	-	-	Anti-HBc: No
Xu, 2018 [29]	92/0	Yes	-	Yes	-	No	Yes	-	Duration of virological remission: Yes

LAM: lamivudine, ETV: entecavir, TDF: tenofovir disoproxil fumarate, c.o.: cut-off, qHBsAg: quantitative HBsAg (IU/mL), HBcrAg: hepatitis B core-related antigen. * only patients with EOT qHBsAg < 200 IU/mL.

**Table 2:** Predictors of clinical relapse after discontinuation of nucleos(t)ides analogues (NAs) in studies including chronic hepatitis B (CHB) patients who remained HBsAg positive at the end of therapy (EOT). Predictors are provided separately for pretreatment HBeAg-positive patients who achieved HBeAg seroconversion and pretreatment HBeAg-negative patients, when available, or mixed patients when separate analyses were not provided.

Study	Patients, n/Cirrhosis	qHBsAg at EOT, IU/mL	Pretreatment HBV DNA, IU/mL	Pretreatment ALT, IU/mL	Age	Sex	Treatment Duration	TDF vs. ETV	Other Markers Tested
**Pretreatment HBeAg-positive patients**									
Chen, 2015 [16]	83/6	No	-	No	Yes	No	No	-	Genotype C, Pretreatment qHBsAg: Yes; Cirrhosis, Bilirubin, Prior LAM, On-treatment/decline of HBsAg levels: No
**Pretreatment HBeAg-negative patients**									
Jeng, 2013 [30]	95/39	No	Yes; c.o.: 2 × 10^5^	-	No	No	No	-	Prior treatment, Pretreatment/EOT qHBsAg: No
Pathwardan, 2014 [31]	33/0	-	No	Yes	No	No	No	-	HBV DNA at 1 month after cessation: Yes
Chen, 2015 [16]	169/29	Yes	No	No	Yes	No	No	-	Cirrhosis, Genotype, Pretreatment /on-treatment/decline qHBsAg: No
Chen, 2018 [19]	90/0	Yes; c.o.: 80	No	-	No	No	No	-	Bilirubin, Genotype, qHBsAg at baseline or on-NA decline: No
Papatheodoridis, 2018 [23]	57/0	No	No	No	No	No	No	No	Detectable HBcrAg at EOT, Pretreatment fibrosis: Yes; Body mass index, Alcohol consumption, Pretreatment AST/albumin/white blood cells/ platelets, liver stiffness at EOT et al.: No
**Pretreatment HBeAg-positive or HBeAg-negative patients**									
Lee, 2016 [32]	44/17	Yes	Yes	-	No	No	No	-	HBeAg: Yes; BMI, DM, HTN, Cirrhosis, PLT, PT, Albumin, Bilirubin, Creatinine: No
Nagata, 2016 [33]	94/0	-	No	Yes	No	No	-	-	Pretreatment qHBsAg, Histol. Fibrosis: No
Hsu, 2019 [34]	135/0	Yes	No	No	Yes	No	No	Yes	EOT ALT, HBcrAg: Yes, Anti-HBe at EOT or pretreatment, HBeAg pretreatment, AST, a-fetoprotein, duration or Viral remission: No
Su, 2018 [27]	100/-‘	Yes; c.o.: 50, 200	-	-	-	-	-	No	Pretreatment HBeAg, CTLA4-nonGG, EOT Anti-HBc, rs3077 non-GG, rs9277535 non-GG, NTCP non-GG: No
Tseng, 2018 [28]	82/0	Yes	-	Yes	Yes	-	-	-	-
Chi, 2019 [35]	100/0	Yes	No	No	No	No	-	No	EOT ALT, HBeAg: No

LAM: lamivudine, ETV: entecavir, TDF: tenofovir disoproxil fumarate, c.o.: cut-off, qHBsAg: quantitative HBsAg (IU/mL), HBcrAg: hepatitis B core-related antigen.

**Table 3:** Predictors of HBsAg loss after discontinuation of nucleos(t)ides analogues (NAs) in studies including chronic hepatitis B (CHB) patients who remained HBsAg positive at the end of therapy (EOT). Predictors are provided separately for pretreatment HBeAg-negative patients, when available, or mixed pretreatment HBeAg-positive or HBeAg-negative patients when separate analyses were not provided.

Study, 1st author, Year	Patients, n/ Cirrhotic	qHBsAg at EOT, IU/mL	Pretreatment HBV DNA, IU/mL	Pretreatment ALT, IU/mL	Duration of on Therapy Virological Remission	Treatment Duration	Age	Sex	Other Markers Tested
**Pretreatment HBeAg-negative patients**									
Hadziyannis, 2012 [36]	33/0	Yes; c.o.: 100	No	Yes	-	-	No	No	EOT ALT: Yes; Previous interferon-alfa: No
Chan, 2011 [37]	53/18	Yes; c.o.: 100	No	-	-	-	-	-	Reduction of qHBsAg from pretreatment to EOT >1 log IU/mL: Yes; Pretreatment qHBsAg: No
Chen, 2014 [14]	105/32	Yes; c.o.: 120	No	No	Yes	-	-	-	-
Hung, 2017 [21]	73/73	Yes; c.o.: 300	-	-	-	-	-	-	Pretreatment platelets: Yes
Jeng, 2018 [38]	691/308	Yes	-	-	-	Yes	-	-	On-NA qHBsAg reduction, Time to undetectable HBV DNA: Yes
Papatheodoridi, 2019 [39]	57/0	Yes; c.o.: 100	No	No	No	No	No	No	Interferon inducible protein 10 at month 1 after EOT: Yes
**Pretreatment HBeAg-positive or HBeAg-negative patients**									
Chen, 2014 [14]	188/44	Yes	Yes	Yes	Yes	Yes	No	No	Cirrhosis, HBeAg, genotype, Pretreatment/on-NA qHBsAg: no
Chi, 2015 [15]	94/27	Yes; c.o.: 100	-	No	-	Yes	-	-	Virological Relapse: No
Nagata, 2016 [33]	94/0	-	Yes; c.o.: 6 log cp/mL	No	-	-	No	No	Pretreatment qHBsAg, Histological fibrosis: No
Yao, 2017 [22]	119 */-	Yes	No	No	No	No	No	No	qHBsAg level or decline during treatment, Cirrhosis, FIB-4, Bilirubin, Baseline qHBsAg: No

ETV: entecavir, TDF: tenofovir disoproxil fumarate, c.o.: cut-off, qHBsAg: quantitative HBsAg (IU/mL). * EOT HBsAg < 200 IU/mL.
